# Improvements in Attention and Cardiac Autonomic Modulation After a 2-Weeks Sprint Interval Training Program: A Fidelity Approach

**DOI:** 10.3389/fphys.2018.00241

**Published:** 2018-03-21

**Authors:** Arilson F. M. de Sousa, André R. Medeiros, Stefano Benitez-Flores, Sebastián Del Rosso, Matthew Stults-Kolehmainen, Daniel A. Boullosa

**Affiliations:** ^1^Post-Graduation Program in Physical Education, Catholic University of Brasilia, Brasilia, Brazil; ^2^Bariatric Surgery Program, Yale-New Haven Hospital, Yale University, New Haven, CT, United States; ^3^Sport and Exercise Science, College of Healthcare Sciences, James Cook University, Townsville, QLD, Australia

**Keywords:** cognition, executive control, high intensity interval training, vagal withdrawal, aerobic fitness

## Abstract

This study aimed to: (1) investigate the influence of a 2-weeks sprint interval training (SIT) program on aerobic capacity, cardiac autonomic control, and components of attention in young healthy university students; and (2) to ascertain whether training fidelity would influence these adaptations. One hundred and nine participants were divided into an experimental (EG) and control (CG) groups. The EG performed a SIT program that consisted of 6 sessions of 4 × 30 s “all-out” efforts on a cycle ergometer, interspersed with active rests of 4 min. The criterion for fidelity was achieving >90% of estimated maximum heart rate (HR) during sprint bouts. After analyses, the EG was divided into HIGH (*n* = 26) and LOW (*n* = 46) fidelity groups. Components of attention were assessed using the Attention Network Test (ANT). Aerobic capacity (VO_2_max) was estimated while the sum of skinfolds was determined. Autonomic control of HR was assessed by means of HR variability (HRV) and HR complexity at rest and during ANT. Both HIGH and LOW significantly increased aerobic capacity, vagal modulation before and during ANT, and executive control, and decreased body fatness after SIT (*p* < 0.05). However, only participants from HIGH showed an increase in HR complexity and accuracy in ANT when compared to LOW (*p* < 0.05). Two weeks of SIT improved executive control, body fatness, aerobic fitness, and autonomic control in university students with better results reported in those individuals who exhibited high fidelity.

## Introduction

Attention is a cognitive process related to the capacity of focusing on an object or task, being fundamental for the selection and retention of stimuli and information that influence most human activities, from the simplest to the complex ones (Rueda et al., 2015). Aerobic exercise, and hence aerobic capacity, have demonstrated to be effective for the improvement of cognitive function over the lifespan (Guiney and Machado, 2013). Previous studies have suggested that greater aerobic capacity is positively related to cognitive function, and more specifically to attention components, such as sustained attention and inhibitory control (Perez et al., 2014; Luque-Casado et al., 2016). However, it is still unknown what aerobic exercise or protocols could be more appropriated for the improvement of attention.

Recently, high-intensity interval training (HIIT) has been suggested to be an efficient alternative for health and physical fitness improvements (Batacan et al., 2017). Sprint interval training (SIT) is a HIIT modality which consists of a number of supramaximal “all out” efforts (≤30 s) interspersed with recovery intervals (~4 min), achieving ~20 min of activity in a single session (Gibala et al., 2012). This training modality has been widely used and demonstrated to positively influence aerobic capacity (Sloth et al., 2013) and various mental (Freese et al., 2014) and cardiometabolic health parameters (Gibala et al., 2012; Sloth et al., 2013), as well as autonomic control of heart rate (HR) (Kiviniemi et al., 2014). However, adherence to these training modalities is questionable since they are exhausting and painful (Hardcastle et al., 2014). Furthermore, another problem refers to individuals' incapacity to achieve the target intensity or to complete training sessions (Hardcastle et al., 2014; Saanijoki et al., 2015). In this regard, a recent study by Taylor et al. (2015) reported that only 58% of bouts met the high-intensity criterion during a 10-week HIIT intervention. This may question the effectiveness of HIIT interventions when the actual exercise intensity does not match the targeted one (i.e., fidelity). Meanwhile, there are no studies verifying the possible influence of SIT interventions on attention components. Therefore, it would be important to look at the potential positive influence of short interventions, such as SIT, for both improvements in physical condition and attention.

Heart rate (HR) variability (HRV) is a simple and non-invasive tool for verification of autonomic nervous system (ANS) activity (Task Force of the European Society of Cardiology, 1996; Schubert et al., 2009; Ernst, 2017). Higher resting HRV is usually related to a greater adaptability and a better recovery of the ANS when responding to stressors (Weber et al., 2010; Castaldo et al., 2015). Furthermore, individuals with higher aerobic capacity exhibit both higher HRV and greater stress tolerance (Hamer and Steptoe, 2007; Luque-Casado et al., 2013). In recent years, there has been a growing effort to better understand the relationship between HRV and cognition (Hansen et al., 2003; Thayer et al., 2009; Luque-Casado et al., 2013). In this regard, some studies have reported that individuals with higher HRV exhibit better results in cognitive tasks (Luque-Casado et al., 2013) including better attention scores (Hansen et al., 2003). This relationship fits very well with the recent neurovisceral model proposed by Thayer et al. (2009) which states that a set of neural structures involved in cognitive, affective and autonomic regulation are related to HRV and cognitive performance. Therefore, there is strong evidence indicating enhanced HRV in individuals with greater aerobic fitness (De Meersman, 1993; Tulppo et al., 2003), and better attention scores in individuals with higher HRV (Hansen et al., 2003; Luque-Casado et al., 2013) and higher aerobic fitness (Perez et al., 2014; Wang et al., 2014). However, to the best of our knowledge, no study has verified the influence of aerobic fitness on both HRV and attention scores after a training period, and more specifically after a time-efficient intervention as SIT.

Thus, the objective of the current study was to verify the influence of a 2-week SIT protocol on aerobic capacity, HRV and attention components in a sample of young healthy university students. Additionally, we aimed to verify if training fidelity would influence these adaptations. It was expected that those individuals who met the high-intensity criterion would exhibit the greater adaptations in all dependent variables.

## Materials and methods

### Participants

Participants were recruited through advertisements placed across the university campus. They were invited and received detailed information about the study and subsequently signed an informed written consent. Participants were instructed not to perform any change in their routines (e.g., incidental physical activity, sleeping hours, nutritional habits, etc.), and not to consume alcohol or caffeine several hours before sessions. The criteria for participation in the study were: not consuming tobacco, medications, supplements, or any drug that could compromise the evaluations; being free of cardiovascular and metabolic diseases, and attention disorders; and not being pregnant. Each participant was informed about the study design and signed a consent form before participating in this study. One hundred and nine participants from a total of 114 candidates were eligible and randomly allocated into experimental (*n* = 89) and control (*n* = 20) groups. After the training and control periods, some participants from both experimental (*n* = 17) and control (*n* = 1) groups withdrew from the study because of lack of time. The local ethics committee of Catholic University of Brasilia, in accordance with Declaration of Helsinki, approved the current study (Protocol number: 43515415.1.0000.0029).

### Procedures

#### Experimental design

The study lasted 4 weeks. On the first day, participants answered a health information and physical activity questionnaire (IPAQ) (Matsudo et al., 2001) and the physical activity readiness questionnaire (PAR-Q). Subsequently, participants were seated in front of a laptop computer in a sound-attenuated room, with the head approximately 50 cm away from the screen, with a cardiac monitor recording their HR. Participants were informed that they would perform the Attentional Network Test (ANT) (Fan et al., 2002) after 10 min of rest. During the following 2 weeks, participants completed the SIT protocol. On the first day of training, an anthropometric evaluation was performed before the training session. Additionally, maximum oxygen consumption (VO_2_max) was estimated with the Åstrand's nomogram after warming up in the first and the last training session. The training consisted of three sessions per week separated by 48 h. The ANT under the same conditions as in the first visit to the laboratory, and an anthropometric evaluation were conducted the last day at the fourth week.

#### Body composition

The measures of body mass (kg) (2096PP, Toledo, São Paulo, Brazil) and stature (cm) (ES-2040, Sanny Medical®, São Paulo, Brazil) were used to calculate body mass index (BMI) for nutritional classification according to the world health organization cut-off points (World Health Organization, 1995). The following skinfolds were measured using a skinfold caliper (Lange Skinfold Caliper®, Beta Technology, California, USA) for determination of sum of skinfolds (SS): pectoral, triceps, subscapular, medial axillary, abdominal, suprailiac, and thigh. All measurements were performed twice and a third measurement was performed if the difference between them was ≥ 2 mm, and the average was calculated and rounded to 0.5 mm (American College of Sports Medicine, 2014).

#### Sprint interval training and aerobic capacity

The SIT protocol was performed for 2 weeks with three sessions per week on a cycle ergometer (Monark Ergomedic 828E, Monark, Vansbro, Sweden) in a periodized fashion. HR was recorded in all training sessions with a HR monitor (RS800CX, Polar Electro Oy, Kempele, Finland).

Firstly, participants performed a 5 min warm-up at 60 rpm with a load of 1-3 kp depending on sex and physical activity (PA) levels [inactive women (1 kp), active women and inactive men (2 kp), and active men (3 kp); Cink and Thomas, 1981]. This warm-up served for estimation of VO_2_max with Astrand's nomogram and the average HR during the last 2-min of HR recordings (Cink and Thomas, 1981; Siconolfi et al., 1982). This is a valid and reliable method for VO_2_max estimation in clinical settings (Cink and Thomas, 1981; Siconolfi et al., 1982; Macsween, 2001).

The first training session served also as familiarization with the cycle ergometer to adjust training loads. Participants of the experimental group performed 3 (sessions 1 and 6) to 4 (sessions 2 to 5) “all out” sprints of 30s with a load corresponding to 7.5% of the body mass (0.075 kp•kg^−1^). The active rest (60 rpm with no load) between sprints and after last sprint (cooling down) lasted 4 min in the first week, and 3 min in the second week. Sessions lasted between 12 and 24 min.

#### Fidelity criterion

Only individuals of the experimental group who completed at least 85% of all programmed bouts (i.e., 22) were considered for analyses. Based on Little et al. (2011) and Taylor et al. (2015), a cut-point of 90% of estimated maximal HR (Tanaka et al., 2001) was used as criterion of compliance. The highest 5-s HR value during every bout of every session was analyzed for compliance assessment.

#### Assessment of attention

The ANT is a valid and reliable test for assessment of attention (Fan et al., 2002) and has been widely used in studies with exercise (Perez et al., 2014; Wang et al., 2016). The ANT is an “arrow test”–a combination between a “flanker test” and a reaction time test. The ANT was developed to analyze the three components that are related to the attention network system: alertness, orientation and execution (Fan et al., 2002). The ANT consisted of one familiarization practice: a block with full feedback and three experimental blocks without feedback. The stimuli consisted of a row of one or five arrowheads pointing leftward or rightward, on gray background. The target was a leftward or rightward arrowhead at the center. Each trial started with a fixation point (+) in the center of the screen lasting 400–1,600 ms. After that, one of four possible cues were presented: no cue, center cue, double cue and spatial cue. Cues consisted of the appearance of an asterisk (^*^) lasting 100 ms presented 400 ms before the appearance of the target (row). The ANT requires the participant to identify the direction of the centrally presented arrow (the target) by pressing one key for the left direction and a different key for the right direction. Participants were instructed to focus on a centrally located fixation cross throughout the task and to respond as quickly and accurately as possible. The three attentional networks efficiencies scores are created using the reaction time (RT) in the cue, congruent and incongruent conditions according to the equations: Alerting effect = RT no cue – RT center cue; Orienting effect = RT center cue–RT spatial cue; Conflict effect = RT incongruent – RT congruent.

#### Evaluation of the autonomic control of heart rate

The R-R intervals were recorded with a cardiac monitor (RS800CX, Polar Electro Oy, Kempele, Finland) at rest during 10 min in a seated position, and the final 5 min were used as baseline measurements (Task Force of the European Society of Cardiology, 1996). Immediately after the baseline determination, participants started the ANT. The last 5 min of R-R recordings during each block were used for HRV analyses. During recordings, participants were asked to breathe freely, and were required not to sniff, sigh, or scratch their throats (Quintana and Heathers, 2014).

The R-R recordings were transferred to custom specific software (Pro Trainer 5 version 5.40.170, Polar Electro Oy, Kempele, Finland). Data was visually inspected; noise and ectopic heart beats were identified (< 3% noise or ectopic beats), corrected and filtered and subsequently exported to a specific software for HRV analyses (Kubios HRV Analysis version 2.2, The Biomedical Signals Analysis Group, University of Kuopio, Finland) (Tarvainen et al., 2014). The following components of HRV were analyzed: standard deviation of R-R intervals (SDNN), root mean square of the successive differences between adjacent normal R-R intervals (RMSSD), the short (SD1) and the long (SD2) axes from Poincarè Plot, sample Entropy (SampEn), and correlation dimension (D2). The above cited HRV parameters were previously examined with exercise (Boullosa et al., 2014; Kiviniemi et al., 2014) and in cognition studies (Luque-Casado et al., 2013; Young and Benton, 2015). Although time domain and Poincaré Plot parameters are very well correlated (Guzik et al., 2007), we decided to present both for allowing comparisons with previous and future studies.

### Statistical analyses

The statistical analysis was performed with SPSS (IBM SPSS Statistics for Windows®, Version 20.0, Armonk, NY, USA). Data are presented as mean ± SD and 95% confidence intervals (95%CI) when appropriate. Normality was assessed with a visual inspection (e.g., skewness, Q-Q plots and box plots) and a Kolmogorov-Smirnov or Shapiro-Wilk test when appropriate. Variables with a non-normal distribution were log-transformed (Ln) for analysis. Comparisons of selected variables between sexes were carried out using *t*-tests for independent samples. A Pearson product-moment correlation coefficient was calculated to assess relationships between selected variables. A two-way analysis of variance (ANOVA) for repeated measures was used with a time effect (before, during and after training) and training effect (experimental group, control group). Effect size (ES) was calculated with Cohen's D. Mauchly's Sphericity was tested, and if sphericity could not be assumed, the greenhouse-Geisser correction was used. The alpha level was set at a *p* < 0.05. All graphics were made with Graph Pad Prism (Version 6.01, Graphpad Software, San Diego, CA, USA).

## Results

**Figure 1:**
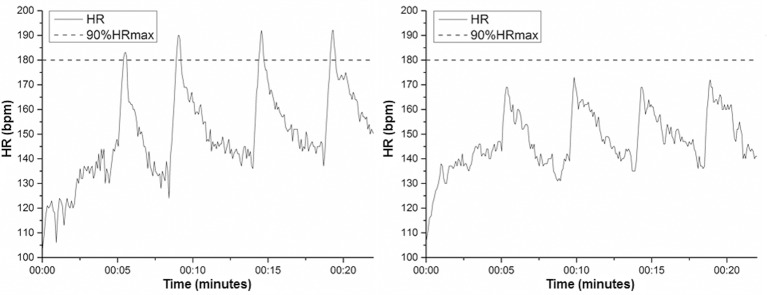
Examples of fidelity analyses with one female participant (20 years old) who achieved the target heart rate (90% of maximum heart rate) during a session **(Left)**, and another female participant of the same age who did not achieve the same target heart rate during the same session **(Right)**.

All participants from the experimental group completed ≥85% of all bouts. However, only 26 participants in the experimental group fulfilled the fidelity criterion. Thus, experimental group was divided into HIGH (*n* = 26) and LOW (*n* = 46) fidelity groups. The estimated maximum HR was 196.5 ± 1.0 bpm (CI95% 194.5–198.6) bpm for HIGH, and 196.5 ± 0.7 (CI95% 195–198) bpm for LOW. The cut-off points (90% of the estimated maximal HR) used as a compliance criterion were the same (176.9 ± 4.6 bpm; CI95% 175–178.7 for HIGH, and 175.5–178.2 for LOW) for both groups. Participants of the HIGH group achieved the target HR in 20.46 ± 1.30 bouts while participants from the LOW group achieved the target HR only in 6.17 ± 6.50 bouts (*p* = 0.000). Peak HR reached by HIGH was 184.16 ± 5.23 (CI95% 182.1–186.5) bpm while LOW reached 170.47 ± 7.49 (CI95% 168.3–172.8) bpm in all sessions (*p* = 0.00). The characteristics of participants in all groups after fidelity analyses are presented in Table 1. An example of fidelity analysis is presented in Figure 1.

**Table 1:** Participants' characteristics of HIGH, LOW, and CG after analysis of fidelity.

	**HIGH (*****n*** = **26)**		**LOW (*****n*** = **46)**		**CG (*****n*** = **19)**	
	**Males *n* = 12**	**Females *n* = 14**	**Males *n* = 25**	**Females *n* = 21**	**Males *n* = 10**	**Females *n* = 9**
Age (years)	23.08 ± 3.96	23.79 ± 6.09	24.12 ± 5.94	22.67 ± 3.80	25.07 ± 4.27	24.33 ± 4.69
METs (MET-min/week)	2,045 ± 1,184	1,329 ± 1,061	2,447 ± 1,544	1,768 ± 1,459	1,562 ± 1,440	1,692 ± 1,124
TV (min/week)	1,530 ± 1,304	2,193 ± 1,601	967 ± 955	1,859.1,152^*^	1,167 ± 1,286	1,860 ± 1,213

*HIGH, high fidelity group; LOW, low fidelity group; CG, control group; METs, MET-min per week: MET level x minutes of activity x events per week (IPAQ), TV (min/week), minutes on screen per week (IPAQ). Data are mean ± SD*.

* *p < 0.05 different from males of the same group*.

Both HIGH and LOW groups, but not the CG, showed significant differences after the training period for SS, VO_2_max, and mean HR during warm-up (*p* < 0.05) (see Table 2).

**Table 2:** Body composition, VO_2_max and HRV measures at rest before and after SIT in HIGH, LOW, and CG.

	**HIGH** ***(n*** = **26)**			**LOW (*****n*** = **46)**			**CG (*****n*** = **19)**		
	**Pre**	**Post**	***d***	**Pre**	**Post**	***d***	**Pre**	**Post**	***d***
BMI (kg·m^−2^)	24.5 ± 3.4	24.5 ± 3.4	0.00	25.0 ± 4.4	25.1 ± 4.4	0.00	23.7 ± 3.9	23.6 ± 3.9	0.00
SS (mm)	148 ± 48	146 ± 48^*^	0.04	144 ± 58	142 ± 58^*^	0.03	124 ± 48	124 ± 48	0.00
VO_2_max (mL·kg^−1^·min^−1^)	36.9 ± 8.6	41.5 ± 6.4^*^	0.63	38.7 ± 9.1	43.5 ± 9.7^*^	0.51	37.9 ± 8.9	38.5 ± 9.5	0.07
HR (average)	141.4 ± 12	133.0 ± 9.4^*^	0.71	137.1 ± 11.4	130.3 ± 9.4^*^	0.65	139 ± 9.9	134.9 ± 7.9	0.40

*VO_2_max, estimated maximum oxygen consumption; HRV, heart rate variability; SIT, sprint interval training; HIGH, high fidelity group; LOW, low fidelity group; GC, control group; BMI, body mass index; SS, sum of skinfolds; HR, the last 2-min heart rate average in warm-up before SIT session. Data are mean ± SD*.

* *p ≤ 0.05 effect of time, d = Cohen's d (effect size)*.

For attention components, both HIGH and LOW significantly improved on the conflict effect (i.e., executive function), but only HIGH exhibited a better result in accuracy scores after SIT (*p* ≤ 0.05). No other changes were observed in any group after the training intervention (see Table 3).

**Table 3:** Attention components before and after SIT during the ANT in HIGH, LOW and CG.

	**HIGH (*****n*** = **26)**			**LOW (*****n*** = **46)**			**CG (*****n*** = **19)**		
	**Pre**	**Post**	***d***	**Pre**	**Post**	***d***	**Pre**	**Post**	***d***
ALERT (ms)	39.3 ± 24.8	39.9 ± 20.7	0.03	41.3 ± 24.4	42.1 ± 24.7	0.03	49.3 ± 20.8	54.2 ± 18.8	0.25
ORIENTING (ms)	41.8 ± 28.8	45.9 ± 18.2	0.17	35.4 ± 25.6	36.3 ± 17.9	0.04	37.7 ± 22.5	39.8 ± 21.3	0.10
CONFLICT (ms)	152.5 ± 63.3	121.7 ± 44.7^*^	0.56	131.2 ± 48.9	106.97 ± 36.79^*^	0.56	129.4 ± 37.7	121.3 ± 30.7	0.24
RT (ms)	668.7 ± 85.1	665.6 ± 84.7	0.03	726.6 ± 109.9	707.31 ± 82.95	0.20	679.0 ± 66.8	663.5 ± 56.8	0.25
ACC (%)	96.2 ± 6.5	98.5 ± 2.6^*^	0.48	95.3 ± 11.7	94.38 ± 12.5	0.08	98.3 ± 1.8	97.7 ± 3.9	0.20

*SIT, sprint interval training; ANT, attention network test; HIGH, high fidelity group; CG, control group; ALERT, alerting component in the ANT test; ORIENTING, orienting component in the ANT test; CONFLICT, conflict effect component in the ANT test; RT, reaction time in the ANT test; ACC, accuracy in the ANT test. Data are mean ± SD*.

* *p ≤ 0.05 time effect, d = Cohen's d (effect size)*.

Analysis of HR and HRV indices before and during the ANT revealed a significant effect of time for all parameters, and a group effect for HR and HR complexity parameters (i.e., SampEn and D2) in HIGH (*p* < 0.05). Indices of vagal modulation (i.e., RMSSD and SD1) and overall variability (i.e., SDNN and SD2), and HR complexity parameters at rest and during the three timepoints of the ANT (before and after SIT) in all groups are presented in Table 4. Of note, while there were only significant differences for HR and HRV complexity measures for HIGH at rest after training (*p* < 0.05), a detailed analysis revealed systematically greater ES for HIGH vs. LOW at all timepoints (ES ranging between 0.15 and 0.29 for HIGH; between −0.30 and 0.20 for LOW).

**Table 4:** HRV measures at rest and the moments in the ANT before and after SIT in HIGH, LOW and Control Groups.

		**HIGH** ***(n*** = **26)**			**LOW (*****n*** = **46)**			**CG (*****n*** = **19)**		
		**Pre**	**Post**	***d***	**Pre**	**Post**	***d***	**Pre**	**Post**	***d***
	Rest	83 ± 10.1	76.3 ± 9.2^*^^#^	−0.69	74.4 ± 13.1	73.2 ± 9.2	−0.09	72.5 ± 11	73.±10.5	0.10
HR (bpm)	1	83 ± 10.2	77.7 ± 8.7^*^^#^	−0.56	75.3 ± 13	73.7 ± 11.7	−0.13	73.6 ± 11.4	75.3 ± 11.4	0.15
	2	83.6 ± 10.2	79.2 ± 9.2^*^^#^	−0.46	76.3 ± 12.8	74.8 ± 12.2	−0.12	73.8 ± 12	76.2 ± 11.4	0.20
	3	83.9 ± 10.5	78.9 ± 9^*^^#^	−0.52	76.2 ± 12	75.3 ± 12.7	−0.07	75 ± 11.9	75 ± 11.9	0.09
	Rest	31.8 ± 13.8	38.8 ± 18.6^*^	0.43	44.3 ± 25.3	50.5 ± 28.6^*^	0.23	46.4 ± 20.3	39.7 ± 16.1	−0.37
RMSSD (ms)	1	32.5 ± 16.8	38.9 ± 17.4^*^	0.37	43.7 ± 23.8	49.2 ± 27.3^*^	0.22	48.6 ± 22.1	40.4 ± 19.3	−0.40
	2	30.1 ± 14.3	37.5 ± 16.9^*^	0.47	41.9 ± 21.7	48.4 ± 26.2^*^	0.27	50.7 ± 25.2	40.6 ± 18.7	−0.46
	3	30.3 ± 13.9	38.4 ± 17.8^*^	0.51	43.8 ± 24.9	47.1 ± 26.6	0.13	46 ± 22	40.5 ± 18	−0.27
	Rest	49.9 ± 16.4	54.8 ± 19.9	0.27	58.3 ± 26.6	67.8 ± 32.8^*^	0.32	66.9 ± 20.8	63.2 ± 24.4	−0.16
SDNN (ms)	1	44.9 ± 13.9	52 ± 17.9^*^	0.41	53.5 ± 23	61.6 ± 29.8^*^	0.30	61.4 ± 20.9	58.3 ± 22.3	−0.14
	2	43.7 ± 15.3	55.7 ± 20.8^*^	0.66	55.5 ± 22.8	65.9 ± 30.7^*^	0.39	65.7 ± 24	64.6 ± 29.1	−0.04
	3	47.8 ± 16.9	57 ± 19.8^*^	0.50	58 ± 27.2	65.9 ± 31.1^*^	0.27	67.2 ± 31.1	63 ± 21.8	−0.16
	Rest	22.4 ± 9.7	27.9 ± 13.1^*^	0.48	31.3 ± 18	35.8 ± 20.3^*^	0.24	31.1 ± 14.7	27.3 ± 12.2	−0.28
SD1 (ms)	1	23.1 ± 11.9	27.6 ± 12.3^*^	0.37	31 ± 16.8	34.8 ± 19.3^*^	0.21	32.5 ± 16	27.5 ± 14.1	−0.33
	2	21.4 ± 10	26.6 ± 11.7^*^	0.48	29.7 ± 15.4	34.3 ± 18.6^*^	0.27	35.9 ± 17.8	28.7 ± 13.3	−0.46
	3	21.3 ± 9.7	27.3 ± 13.1^*^	0.52	31 ± 17.6	33.4 ± 18.8	0.13	32.6 ± 15.6	28.7 ± 12.8	−0.27
	Rest	67.9 ± 21.5	75.8 ± 32	0.29	75.8 ± 34.3	88.4 ± 43^*^	0.32	88 ± 26.7	82.5 ± 36.9	−0.17
SD2 (ms)	1	57.9 ± 17.5	67.7 ± 23.5^*^	0.47	68.7 ± 28.9	79.5 ± 38.1^*^	0.32	78.3 ± 25.4	75.4 ± 29.2	−0.10
	2	57.9 ± 19.9	74.6 ± 27.4^*^	0.70	72.4 ± 29.4	86.1 ± 40.3^*^	0.39	83.2 ± 30.8	86.5 ± 39.4	0.09
	3	63.9 ± 22.6	75.6 ± 24.6^*^	0.50	75.4 ± 35.0	86.5 ± 40.6^*^	0.29	87.5 ± 41.1	84.1 ± 28.4	−0.10
	Rest	1.35 ± 0.24	1.43 ± 0.28^*^^#^	0.31	1.54 ± 0.27	1.47 ± 0.35	−0.22	1.41 ± 0.39	1.4 ± 0.28	−0.03
SampEn	1	1.54 ± 0.33	1.62 ± 0.28	0.26	1.67 ± 0.22	1.59 ± 0.31	−0.31	1.56 ± 0.32	1.49 ± 0.29	−0.23
	2	1.54 ± 0.27	1.6 ± 0.49	0.1	1.57 ± 0.28	1.51 ± 0.34	−0.19	1.61 ± 0.3	1.45 ± 0.25	−0.58
	3	1.44 ± 0.27	1.51 ± 0.27	0.26	1.57 ± 0.25	1.5 ± 0.3	−0.25	1.5 ± 0.21	1.45 ± 0.23	−0.23
	Rest	2.44 ± 1.25	2.95 ± 1.27^*^^#^	0.41	2.98 ± 1.35	2.79 ± 1.39	−0.14	3.02 ± 1.11	3.02 ± 1.16	0
D2	1	2.63 ± 1.43	3.05 ± 1.43	0.29	2.87 ± 1.57	3.03 ± 1.45	0.11	3.27 ± 1.06	3.05 ± 1.23	−0.19
	2	2.66 ± 1.52	2.91 ± 1.28	0.18	2.84 ± 1.39	2.87 ± 1.35	0.02	3.13 ± 0.96	3.11 ± 1.29	−0.02
	3	2.64 ± 1.49	3 ± 1.13	0.27	2.75 ± 1.46	3.02 ± 1.28	0.20	3.26 ± 1.16	3.2 ± 1.05	−0.05

*HR, heart rate; SDNN, standard deviation of R-R intervals; RMSSD, root mean square of the successive differences between adjacent normal R-R intervals; HRV, heart rate variability; SIT, sprint interval training; HIGH, high fidelity group; LOW, low fidelity group; CG, control group; SD1, short-term beat-to-beat R-R variability from the Poincaré plot; SD2, long-term beat-to-beat variability from the Poincaré plot, SampEn, sample entropy; D2, correlation dimension. Data are mean ± SD*.

* *p < 0.05 time effect*,

# *p < 0.05 HIGH vs. LOW, d = Cohen's d (effect size)*.

There were no significant correlations between dependent and independent variables for any group.

## Discussion

To our knowledge, this is the first study showing an effect of SIT on attention, aerobic fitness, and cardiac autonomic control in young adults, controlling for the potential influence of participants' fidelity to training. By taking this approach, we observed that only 2 weeks of SIT resulted in significant increases in several outcomes of interest. More importantly, those participants meeting fidelity criteria showed better ANT accuracy and HRV complexity measures compared with those with low training fidelity.

As shown in previous studies (Gibala et al., 2012; Sloth et al., 2013; Kiviniemi et al., 2014), SIT programs can improve aerobic fitness and cardiac autonomic control in the short term. However, there are no previous reports on the effects of a SIT program on outcomes of attention demanding tasks. Previous studies reported improvements in attention with an acute session (Alves et al., 2014; Ma et al., 2015), and one study reported small to moderate non significant improvements in executive function after 8 weeks of HIIT (Costigan et al., 2016). Although differences in the training protocols (HIIT vs. SIT) make comparisons rather difficult, the combined results of the previous and the current studies suggest that aerobic fitness could be positively related to better performance in different attention- demanding tests (Perez et al., 2014; Luque-Casado et al., 2016; Wang et al., 2016), especially those requiring executive control. In fact, it has been suggested that brain structures associated with executive control (i.e., prefrontal and cingulate cortex), are relatively sensitive to physical exercise stimuli and seem to be more active in individuals with higher aerobic fitness (Guiney and Machado, 2013). As such, our observation of an increase in aerobic fitness, combined with higher scores in the conflict measure of the ANT in the EG compared to the CG, seems to support the relationship between aerobic fitness and attention. Meanwhile, as it is also expected that SIT would influence anaerobic capacity (Hazell et al., 2010), it is still unknown if there is any link between improvements in anaerobic capacity (not recorded in the current study) and attention. However, this aspect may not be relevant since there was not any difference in adaptations between sexes in the current study.

Higher aerobic fitness has been previously related to a better cardiac autonomic control (Hamer and Steptoe, 2007; Luque-Casado et al., 2013). This observation was also the case for the EG in the present study, as this group experienced an increase in aerobic fitness and HRV, both at rest and during the ANT, after 2 weeks of a SIT program. Thus, the higher HRV after the SIT training program suggests an improvement in cardiac autonomic control. In addition to changes in HRV, those participants in the EG also showed an enhanced executive control after the SIT program. Based on the information available, it could be argued that these results can be linked to the neurovisceral integration theory proposed by Thayer et al. (2009), which suggests that a set of neural structures involved in cognitive, affective, and autonomic regulation are related to HRV and cognitive performance. More precisely, the behavior of subcortical areas, such as the amygdala, would be inhibited by pre-frontal cortical projections, which in time will favor higher inhibitory control and greater cognitive flexibility during, for instance, the conflict stimuli provided by the ANT test. Consequently, although further research is needed to draw any definitive conclusion, it could be hypothesized that any improvement in aerobic fitness, as observed following a 2-week SIT program, can have positive effects on cognition by enhancing autonomic regulation via complex neurovisceral regulation.

One potential confounding factor to be considered is body composition and its effects on HRV and cognition. For instance, Esco et al. (2011) reported a higher cardiac autonomic control related to a decrease in SS that was independent of changes in aerobic fitness in a group of young adults. In addition, there are observations of a better cognitive performance in individuals with lower body fat percent (Tikhonoff et al., 2015). In the present study we found a reduction in SS in the EG after the training program, although the effect size was small (*d* = 0.04). Of note, the participants of the present study were asked to maintain their usual dietary routines to restrict possible additional interferences. Consequently, while the potential contribution of the skinfold reduction on HRV cannot be ruled out, it is likely that such effect was negligible. Nevertheless, given that longer training periods (i.e., ~12 weeks) can result in significant changes in body composition (Batacan et al., 2017), further studies are needed to gain a deeper insight on the relationship between body composition and cognition.

An important finding of current study was the use of fidelity criterion as a composite measure of both attendance and compliance. From this analysis, it was possible to verify differences in measures of HRV and attention within the EG that would have been missed otherwise. In this regard, Montero and Lundby (2017) recently reported that individuals classified as non-responders to a cardiorespiratory fitness program became “responders” by increasing the dose of exercise. Thus, the different response of the internal load (HR) between HIGH and LOW when performing the same external workload (kp and rpm), could have enforced greater demands on the aerobic metabolism (Montero and Lundby, 2017) leading to different adaptations between groups. However, as previously commented, there were no significant differences between fidelity groups for changes in aerobic fitness after SIT. Therefore, it would be suggested that further improvements of HIGH could be associated with factors other than aerobic fitness. It is also worth noting that only HIGH displayed improvements in complexity measures of HRV (i.e., SampEn, and D2) when compared with LOW. These complexity measures have been previously related to stress tolerance (Schubert et al., 2009) and executive control (Young and Benton, 2015). Therefore, the increase in HRV complexity measures observed in the present study would likely reflect a higher attentional ability and stress resilience to stressors such as the conflict component of the ANT test. Meanwhile, it is unknown how the different adaptations of HIGH and LOW after the same SIT program, could be related to different autonomic adaptations. In fact, it is interesting to note the significant reduction in HR only in HIGH after the training period, which may be linked to changes in complexity HRV measures. One possible explanation could be the higher HR acceleration of HIGH during training sessions, which is related to a greater vagal withdrawal during exercise (Bellenger et al., 2016). Of note, it should be pointed out that the external load and the cut-off points for fidelity criterion were the same for both HIGH and LOW. Therefore, it may be suggested that similar physical demands for both groups resulted in different autonomic responses during exercise (i.e., higher vagal withdrawal in HIGH) and therefore in greater autonomic adaptations (i.e., higher HRV complexity measures) that favored an improvement in more aspects of attention. Further studies should elucidate the mechanisms behind these different autonomic adaptations between groups, including fidelity analysis as a venue to ascertain the dose-response relationship for various exercise training approaches. In addition, as both groups exhibited different but non-significant values for HR and HRV parameters at rest before SIT (see Table 4), further studies should verify the potential influence of initial HR and HRV values on autonomic adaptations after different SIT and HIIT programs, while controlling a possible anticipatory effect (i.e., vagal withdrawal leading to a higher HR at rest) that could not be excluded in HIGH as baseline measures were determined before performing the ANT test.

One interesting aspect is the important difference in number of participants between HIGH and LOW groups (26 vs. 46, respectively). While this difference could not be explained mechanistically, it would be reasonable if we consider the previous studies of Little et al. (2011) and Taylor et al. (2015) in which participants performed longer exercise bouts of 60 and 45 s, respectively. As we determined the fidelity criterion based on these previous studies (Little et al., 2011; Taylor et al., 2015), it would be expected that more individuals than in those previous studies would not achieve the target HR; our participants performed shorter sprints of 30 s (Tschakert and Hofmann, 2013). Therefore, further studies should elaborate on specific target HR and other internal workload parameters for fidelity criteria. These would likely vary by the duration of exercise and other external workload parameters (e.g., recovery, intensity) for different populations. In this respect, estimating maximum HR for the use of a fidelity criterion could be a potential limitation. However, the formula currently used (Tanaka et al., 2001) is valid for this population, and confidence intervals for estimated and actual HR values reveal an absence of overlapping that reinforces the validity of this approach. Further studies with observed values are warranted to shed light on this issue.

### Study limitations

Despite several positive aspects of the present study, some limitations should be acknowledged. For instance, the use of indirect methods to assess aerobic fitness, despite its validity and ecology, could restrict the validity of our conclusions. However, changes in aerobic fitness estimated in the current study are comparable to previous reports with similar protocols. In addition, although participants were strongly encouraged to maintain their usual lifestyle routines, incidental physical activity was not controlled, which could be a potential confounding factor. Another potentially confounding factor may be the absence of information regarding the sports practiced by participants and their training background, factors that could also influence the results. Furthermore, controlling the dietary intake would also provide more information regarding the effect of SIT on body composition changes. Future studies should elaborate on the control on these and other factors involved in physical and cognitive performances.

## Conclusions and future perspectives

Our results showed that only 2 weeks of SIT could be an effective technique to improve aerobic fitness and cardiac autonomic control, with potentially positive effects for enhancing executive control in healthy young adults. Individuals with higher aerobic fitness and greater autonomic control are also more resilient to stressful situations, which in time can favor psychological and cardiovascular health. Also, young adults in the apex of their productive life could benefit from improvement of executive control, an important feature of daily cognitive activities. From a dose-response perspective, consideration of a fidelity criterion would help for better control of the positive impact of these exercise interventions. Compared with previous studies with longer interventions of varying lengths, our results for attention components are clinically relevant. We observed moderate ES (0.48 to 0.56) that were higher than the ES reported by Hillman et al. (2014) with children (0.34) but lower than the ES (0.77) reported by Vaughan et al. (2014) with elderly women. This is very important given that our participants were healthy active young adults; therefore., greater ES would be expected in sedentary or unhealthy participants. Future studies are needed, which should include monitoring dietary intake and incidental physical activity to provide a better understanding of training effects. Moreover, the use of longer training programs and even other types of HIIT would help to gain a deeper insight into the effects of exercise on attention and cardiac autonomic control. Given that previous studies have suggested a link between BDNF levels and exercise intensity (Marquez et al., 2015), and between BDNF and performance during attention tests (Leckie et al., 2014), future studies analyzing the influence of different SIT and HIIT programs on BDNF levels are warranted.

## Author contributions

AdS and DB designed the study. AdS, AM, and DB conducted the study and analyzed the data. SB-F, SD, MS-K, and DB contributed to the interpretation of data. AdS wrote the first draft of the manuscript. DB, SD, SB-F, and MS-K contributed with comments and ideas that improved the first draft. AdS, AM, SF, SD, MS-K, and DB revised and approved the final version of the manuscript.

### Conflict of interest statement

The authors declare that the research was conducted in the absence of any commercial or financial relationships that could be construed as a potential conflict of interest.
